# Editorial: Management and Treatment of Pilonidal Disease: 189 Years After Mayo

**DOI:** 10.3389/fsurg.2022.950793

**Published:** 2022-06-14

**Authors:** Gaetano Gallo, Marco Milone, Marco La Torre, Luigi Basso

**Affiliations:** ^1^ Department of Surgical Sciences, La Sapienza University of Roma, Roma, Italy; ^2^ Department of Clinical Medicine and Surgery, “Federico II” University of Naples, Naples, Italy; ^3^ Coloproctology Unit, Salvator Mundi International Hospital, UPMC University of Pittsburgh Medical Center, Roma, Italy; ^4^ “Pietro Valdoni” Department of Surgery, Policlinico Umberto I, Sapienza University of Roma, Roma, Italy

**Keywords:** pilonidal disease, minimally invasive procedures, endoscopic procedures, patients satisfaction, pilonidal sinus disease

**Editorial on the Research Topic**

***Management and Treatment of Pilonidal Disease: 189 Years After Mayo***

Pilonidal Disease (PD) was first scientifically reported 189 years ago by Herbert Mayo, back in 1833, as a sinus containing hair follicles located in the sacrococcygeal region of a female patient (1).

“Pilonidal Disease” is probably a more comprehensive and appropriate term to describe this condition, as a cyst and/or a fistula can both be detected in the individual patient. Indeed, PD more commonly affects the sacrococcygeal area, but, other sites, such as umbilicus, suprapubic area, external genitalia (penis, scrotum, clitoris), axilla, sternum, breast, intermammary area, interdigital clefts (occupational disease: hairdressers i.e., “barber’s disease”) may rarely be involved, supporting the theory of the acquired nature of this condition.

Males are more commonly affected than females, with a 3:1 ratio, especially from puberty until the early 30’s (2). Hirsutism in the natal cleft and buttocks with excess keratin in the hair follicle is a common feature, together with sedentary lifestyle and obesity and contour of the natal cleft (deep, overhanging areas, dips). Previous over-employment of steroids (i.e., asthma) is sometimes reported, as well as risky occupations (“jeep disease” of United States Army). Even if not hereditary, some factors such as shape, size, strength and scaliness of patient’s hair, high degree of friction, pressure, or traumatic injury to tailbone, should be considered.

According to Bascom’s widely accepted theory of 1980, PD originates from hair follicles of the natal cleft (3). Keratin initially occludes a stretched follicle, which becomes inflamed and breaks into adjacent adipose tissue, thus forming a pilonidal micro-abscess. If the micro-abscess drains towards the outside, inflammation subsides, and the mouth of the follicle reopens. The remnant of the follicle and micro-abscess cavity forms a chronically draining pilonidal sinus.

However, often, loose, and vagrant hairs from the region gather in the natal cleft, encouraged by the contour of the area and by the dynamic forces involved, prevent the sinus cavity to heal promptly with a consequent true epithelium-lined fistula.

PD, depending on the stage of the condition, clinically presents as a painful and redness lump, more or less tender with chronic secretions, possible fever and generalized malaise, fistulous track(s), recurring infections and/or abscesses, one or more median and/or lateral primary pits.

Malignant degeneration of PD is possible but extremely rare. In 1994, Davis et al. (4) presented three new cases of degenerated PD with a review of the world's previously published 41 cases. Of these total 44 cases, 36 were squamous cell carcinoma. All cases occurred in long-standing PD, with the mean duration of PD being 23 years. Five of six patients presenting with inguinal metastases died within 16 months. Four patients received adjuvant radiotherapy, one received adjuvant chemotherapy, and one patient received both adjuvant chemo- and radiotherapy. Six patients with recurrence received potentially curative resection, with three patients surviving >10 years with no evidence of disease. The authors recommended adjuvant chemo- and radiotherapy as a new modality treatment to decrease local recurrence rate (4).

In this context, Vertaldi et al. focused their article on the importance of histopathological examination in the prevention of malignant degeneration in patients with PD. In particular, the authors showed that even in the case of minimally invasive endoscopic procedures it is possible to harvest a sample in order to rule-out the diagnosis of carcinoma. Interestingly, none of the 45 patients included in the study had evidence of malignancy.

The choice of the ideal treatment is still controversial although several procedures have been described (5–8). The decision should be based on both the presentation of the disease, i.e., de novo or recurrent, with or without the presence of an abscess, and surgeon’s experience as well as according to the patient’s desire to definitively resolve his/her condition. In fact, considering the embarrassment and the strong psychological stress caused by the disease, Segre pointed out the importance of a shared decision with the patient based on a thorough explanation of the advantages and disadvantages of each surgical approach. Moreover, a proper post-operative care, which can only be implemented by the patient after a complete and clear understanding of the disease, is a fundamental step in the prevention of recurrence.

Guidelines for diagnosis and treatment of PD should be developed and regularly updated, parallel to the development of new ideas and technology (2, 9, 10). Nowadays, it is evident how minimally invasive endoscopic procedures are becoming increasingly popular. These seem to achieve good results, in terms of low recurrences (albeit always present, except in “dreamland”), low or no pain, quick healing, and prompt return to daily activities. Even minimally invasive procedures, however, may present post-operative complications (11), and they should be performed by dedicated surgeons with some degree of experience, as they have proved to be more successful in the hands of dedicated surgeons (8).

In confirmation of this latter statement, Gallo et al. reported a tertiary care academic center experience including 32 non-consecutive patients with PD with a mean number of external openings of 2.41 (1–4) ± 1.04 who underwent endoscopic pilonidal sinus treatment (EPSiT). The overall success rate was 87.5% (28/32 patients) with a mean follow-up period of 22 (4–42) ± 11.49 months. The results were very promising and in line with those reported in the literature.

However, the length of the follow-up does not allow us to draw definitive conclusions even if we are close to that 5–10-year follow-up target that will help to definitively establish the effectiveness of minimally invasive procedures (12, 13) and probably, as suggested by Manigrasso and colleagues to extend the indication not only to a limited PD but also for the treatment of complex cases.
